# Supplementary material for: Predictors of Changes in Physical Activity and Sedentary Behavior during the COVID-19 Pandemic in a Turkish Migrant Cohort in Germany
Source: Int J Environ Res Public Health. 2021 Sep 14;18(18):9682. doi: 10.3390/ijerph18189682 (PMC8472770; doi:10.3390/ijerph18189682)
Supplement: Supplementary file 1 [file ijerph-18-09682-s001.zip › ijerph-1357862-supplementary.pdf]

Table S1. Contingency coefficients* of different physical activities.

	PA at Work	PA at Home	Leisure-Time PA	Sporting PA	PA as Transport
PA at work	1	0.5	0.6	0.6	0.6
PA at home	-	1	0.4	0.54	0.4
Leisure-time PA	-	-	1	0.7	0.7
Sporting PA	-	-	-	1	0.7
PA as transport	-	-	-	-	1

*0–0.2 weak association, >0.2–0.6 moderate association, >0.6–1 strong association (TSC Germany (Hrsg), 2014).

Table S2. Factors associated with reduced physical activity (PA) and/or increased sedentary behavior (SB). Univariable regression analysis for the outcome Less activity/more sedentary behavior.

	PA at Work	PA at Home	Leisure-Time PA	Sporting PA	Active Transport	Sedentary Behavior
			Less PA*			More SB**
			OR (95% CI)			
Socio-demographics						
Age difference	−0.1 (−5.5; 5.3)	3.5 (−5.0; 12.0)	−0.3 (−4.9; 4.4)	−1.0 (−5.7; 3.7)	−0.7 (−5.4; 4.0)	1.8 (−3.1; 6.7)
Sex						
female (Reference)	1	1	1	1	1	1
male	0.6 (0.3;1.6)	0.4 (0.1;2.3)	1.1 (0.5;2.4)	0.6 (0.3;1.3)	0.4 (0.2;0.9)	0.7 (0.3;1.6)
Education						
<10 years (Reference)	1	1	1	1	1	1
10-12 years	0.8 (0.3;2.5)	0.6 (0.1;2.9)	2.3 (0.9;6.0)	1.3 (0.5;3.3)	0.7 (0.3;1.9)	1.5 (0.6;4.0)
>12 years	0.7 (0.2;2.3)	No data available	0.7 (0.2;2.2)	1.3 (0.4;4.0)	0.8 (0.3;2.5)	1.3 (0.4;4.2)
Employed						
Yes (reference)	1	1	1	1	1	1
No	0.8 (0.3;2.1)	0.8 (0.2;3.6)	0.8 (0.4;1.8)	1.0 (0.4;2.2)	1.1 (0.5;2.6)	1.3 (0.6;3.0)
Questionnaire language						
German (Reference)	1	1	1	1	1	1
Turkish	0.9 (0.3;2.3)	1.0 (0.2;4.5)	1.7 (0.8;3.8)	1.5 (0.6;3.4)	3.2 (1.3;7.5)	1.2 (0.5;2.7)
Own migration experience						
Yes (Reference)	1	1	1	1	1	1
No	0.8 (0.2;2.5)	No data available	2.5 (0.8;7.7)	1.1 (0.4;3.3)	0.8 (0.3;2.6)	1.0 (0.3;3.0)
Health status						
Normal or overweight (Reference)	1	1	1	1	1	1
obesity	1.2 (0.4;3.4)	0.2 (0.03;2.2)	1.9 (0.8;4.5)	1.8 (0.7;4.4)	5.1 (2.0;13.4)	1.5 (0.6;3.7)
Smoking behavior						
Smokers (Reference)	1	1	1	1	1	1
Never or Ex-Smoker	1.0 (0.4;2.8)	0.4 (0.1;1.8)	0.6 (0.3;1.4)	1.4 (0.6;3.4)	0.9 (0.4;2.2)	1.0 (0.4;2.5)
At least 150min/week physically active						
Yes (Reference)	1	1	1	1	1	1
no	1.45 (0.39;5.41)	1.24 (0.13;11.82)	1.57 (0.54;4.58)	4.94 (1.54;15.84)	1.14 (0.39;3.38)	6.56 (1.38;31.23)
Subjective health status						
Good, very good, excellent (Reference)	1	1	1	1	1	1

Not so good, poor	1.82 (0.60;5.51)	2.27 (0.50;10.38)	1.02 (0.40;2.60)	1.08 (0.41;2.86)	1.18 (0.45;3.08)	1.31 (0.50;3.45)
Depressive symptoms						
No (PHQ-9 score < 10) (Reference)	1	1	1	1	1	1
Yes (PHQ-9 score ≥ 10)	2.33 (0.83;6.54)	0.89 (0.16;4.87)	1.55 (0.64;3.73)	1.49 (0.60;3.73)	2.38 (0.96;5.88)	2.37 (0.95;5.91)

*Less or much less vs. no change, more or much more; ** More or much more vs. no change, less or much less.
